# Familiarity of teaching skills among general practitioners transfer training trainers in China: a cross-sectional survey

**DOI:** 10.1186/s12909-023-04945-3

**Published:** 2023-12-12

**Authors:** Kang An, Ruohan Zhang, Binlu Zhu, Liyun Liu, Jiayu Tang, Yiru Ma, Zengxiang Wu, Lin Zhang, Yi She, Dan Luo, Caizheng Li, Heting Li, Yuehua Ma, Weichang Shi, Qiaoli Su, Shuangqing Li

**Affiliations:** 1 General Practice Ward/International Medical Center Ward, General Practice Medical Center, National Clinical Research Center for Geriatrics, West China Hospital, Sichuan University, Chengdu, Sichuan China; 2 West China School of Public Health, Sichuan University/West China Fourth Hospital, Sichuan University, Chengdu, Sichuan China; 3 Department of Pediatrics, West China Second University Hospital, Sichuan University, Chengdu, Sichuan China; 4 General Practitioners’ Training Center of Sichuan Province, Chengdu, Sichuan China; 5 Nanchang University Queen Mary School, Nanchang, Jiangxi China; 6 University of Copenhagen, Copenhagen, Denmark; 7 Fang-cao Community Health Service Center, Chengdu, Sichuan China; 8 Jin-cheng Community Health Service Center, Chengdu, Sichuan China

**Keywords:** General practice, Primary care, Teaching skill, Familiarity, Trainer, Transfer training

## Abstract

**Background:**

The insufficient number of general practitioners (GPs) is a major challenge facing China’s healthcare system. The purpose of the GP transfer training programme was to provide training for experienced doctors to transition to general practice. However, research on the competencies of GP transfer training trainers in teaching skills in China is limited. This cross-sectional study aimed to examine the baseline familiarity with teaching skills among Chinese GP transfer training trainers.

**Methods:**

An online survey was conducted among trainers who participated in the 2021 Sichuan Province General Practice Training Trainer Program. The survey collected data on participants’ characteristics and familiarity with 20 skills in three essential teaching knowledge areas: the core functions of primary care (five questions), preparation for lesson plan (four questions), and teaching methods (11 questions).

**Results:**

In total, 305 participants completed the survey. Familiarity rates were generally low across all three essential teaching knowledge areas. No significant differences were observed in familiarity rates between the tertiary and secondary hospitals.

**Conclusion:**

This study revealed gaps in the teaching skills of GP transfer training trainers in China. These results suggest the necessity for targeted training programs to enhance the teaching skills and competencies of trainers.

**Supplementary Information:**

The online version contains supplementary material available at 10.1186/s12909-023-04945-3.

## Introduction

**Fig. 1:**
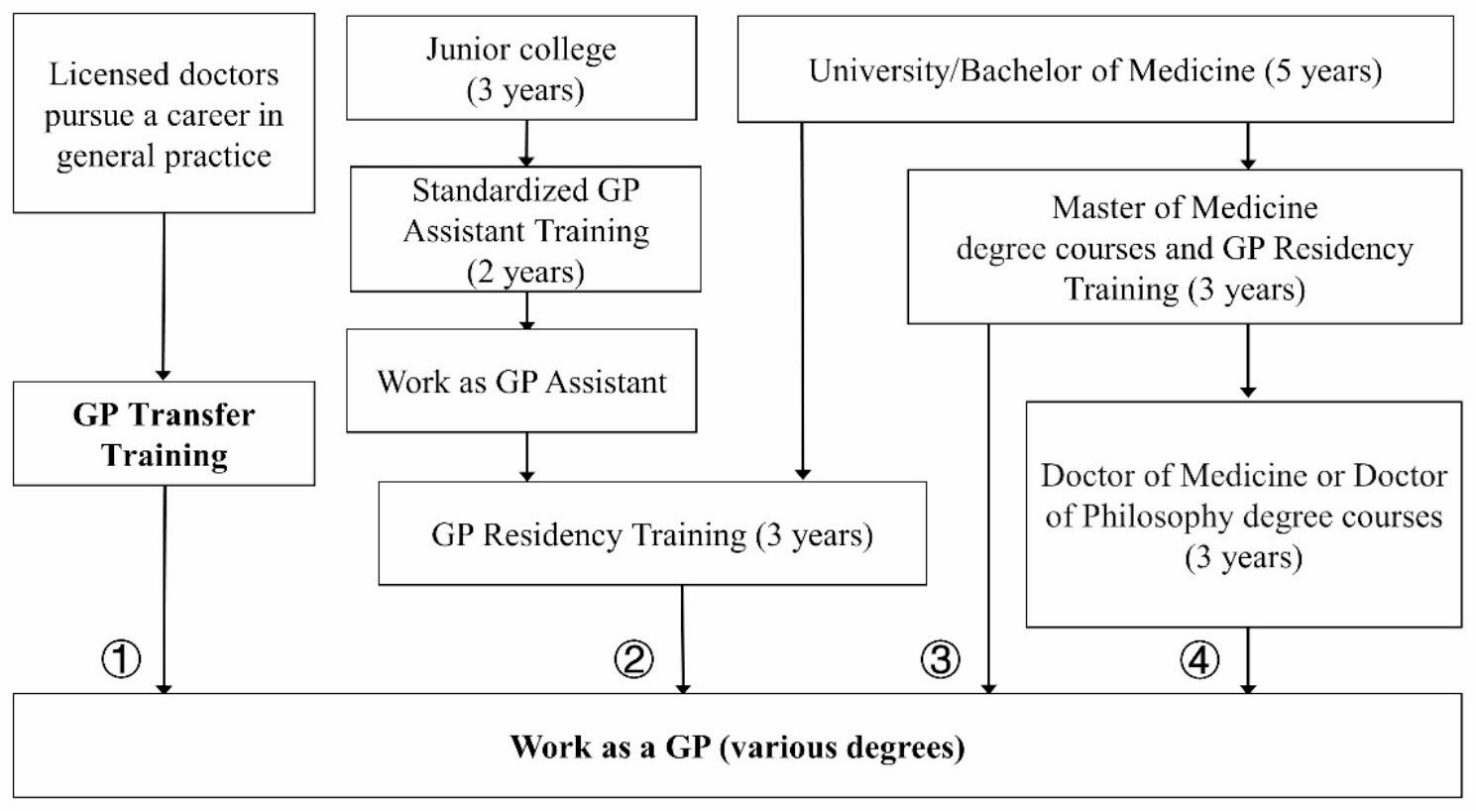
Key Pathways to Becoming General Practitioners in China [7] *Note*: GP, general practitioner

The shortage of general practitioners (GPs) is a significant challenge in China’s healthcare system. After more than 20 years, the Chinese government implemented educational reforms aimed at increasing the number of GPs in the country [1–3]. In China, the number of GPs per 10,000 population increased from 2.9 in 2020 to 3.28 in 2022 [4]. However, a substantial disparity persists when compared to developed countries such as Austria (7.5), Ireland (8.6), Italy (7.0), and the United Kingdom (8.1) in 2021 [5]. Recent projections estimate that the number of GPs in China will reach 550,000 by 2025, with a ratio of 3.93 GPs per 10,000 population [6]. However, this is still some distance away from the national target of 5 GPs per 10,000 people [7]. To address this shortfall, the Chinese government has implemented multiple pathways for becoming a GP [7, 8]. Figure 1 provides a comprehensive overview of the four major pathways to becoming a GP in China.

Currently, GP transfer training is an important training pathway (see details in the additional file 1). Transfer trainers play a crucial role in GP transfer training programs. They are responsible for guiding clinical practitioners through the transition to general practice, which involves developing the necessary knowledge, skills, and attitudes. Requirements to become trainers in GP transfer training include [9]: (1) Planning to undertake training responsibilities in GP training, practice, or theoretical teaching. (2) Holding a bachelor’s degree or higher. (3) Attaining the title of attending physician or higher in professional titles. (4) Possessing substantial clinical teaching experience. (5) Completing the training assessment and obtaining a certificate of qualification for GP Transfer Training trainers. The total duration of the training is 8 weeks. The quality of learning in a clinical environment is largely dependent on the pedagogical and organisational support afforded to them [10, 11]. Therefore, it is important to ensure that transfer trainers are adequately trained and have a sufficient understanding of the key elements of primary care and teaching skills.

Owing to the limited number of healthcare institutions with general practice in China, the rotation task of general practice can be completed in internal medicine [12, 13]. This results in many trainers coming from specialist fields rather than general practice [14]. Until recently, few studies have been conducted on the competence of teaching skills among GP transfer training trainers in China. Most previous studies have emphasised the requirements of trainees. This cross-sectional study examined the degree of baseline familiarity with teaching skills among Chinese GP transfer training trainers.

## Methods

### Study design

This study involved an online survey of trainers who participated in the 2021 Sichuan Province General Practice Train-the-Trainer Program. It reports the analysis of data collected during the training preparation phase, measuring trainers’ baseline knowledge of teaching skills during transfer training. Data were collected from 10th April to 25th May, 2021. The survey was conducted during the training-preparation phase. The inclusion criteria were as follows: (1) Participation in GP transfer training as a trainer. (2) The participants who were willing to participate in the study.

### Data collection

The training participants were invited to complete the questionnaire within a two-week period by accessing a link provided in a text message. The Questionnaire Star platform (https://www.wjx.cn) was used to collect data from participants. Two reminder text messages were sent. They were asked to complete an optional pre-training survey before commencing the training. To avoid information bias, participants were asked to complete survey questionnaires anonymously.

### Quality control

We implemented rigorous quality control measures to ensure data quality. Respondents were limited to submitting their survey responses only once and were unable to make any edits after submission. Additionally, all survey items were required to be completed before submission, and survey data containing logical errors were eliminated.

### Description of questionnaire

Based on previous research findings, we developed a self-assessment questionnaire on teacher preparation [15, 16]. Some questions were drawn from the training programme for GPs transfer (2019 version) [17]. An advisory panel comprising GP trainers with over ten years of experience and policymakers assessed the questionnaire for length (less than 15 min), clarity, practicality, and comprehensibility. The questionnaire underwent iterative rounds of review and validation. A pilot study involving twenty GPs was conducted to test the content validity, evaluating the feasibility and acceptability of the questionnaire. They indicated that the questionnaire was relatively clear and easy to complete. This tool was used to evaluate the gaps in essential knowledge, identify training priorities, and inform curriculum development. The questionnaire was comprised of two sections: characteristics (seven questions) and familiarity of teaching skills, (Please refer to additional file 2: A Teaching Skills Questionnaire for General Practitioners Transfer Training).

First, participants’ characteristics included age, sex, years of practice, education level, and workplace, whether they had clinical teaching experience, and whether they worked in general practice. Section 2 asked about 20 skills in three essential teaching knowledge areas: the core functions of primary care (five questions), preparation for lesson plans (four questions), and teaching methods (11 questions). The core functions of primary care encompassed people-centred care, comprehensiveness, continuity, coordination, and first contact accessibility [18, 19]. Respondents indicated their level of familiarity using a five- level Likert scale (1 = not at all familiar, 2 = slightly familiar, 3 = somewhat familiar, 4 = moderately familiar, and 5 = extremely familiar). Higher levels of familiarity indicated a lower need for training.

### Ethics

This study was approved by the Ethics Committee of West China Hospital, Sichuan University, Chengdu, China (No. 2021 − 1735). All participants were informed of the study’s purpose and that their data would remain anonymous and confidential.

### Data analysis

Descriptive statistics were used to analyse the quantitative data. Comparisons between two groups were performed using the chi-square test. Statistical significance was set at P < 0.05 (two-tailed) indicated statistical significance. Microsoft Excel was used to create graphical representations. All data were analysed using SPSS software (version 24.0; IBM Corporation).

To analyze training needs based on different levels of familiarity, the established criteria for distinguishing between “familiar” and “relatively unfamiliar” were as follows: respondents selecting “moderately familiar” and “extremely familiar” were categorized as “familiar,” while those choosing “not at all familiar,” “somewhat familiar,” or “slightly familiar” were considered “relatively unfamiliar.” Familiarity rates were defined as the proportion of response options combining “moderately familiar” or “extremely familiar,” indicating no necessity for training in the specific area.

## Results

### Demographic characteristics

The response rate was 94.1% (305/324) among total training registrants. After logical testing, 305 questionnaires (100%) were included in the analysis. Respondents were recruited from 150 county-level training bases (comprehensive county-level hospitals) in 21 cities. Table 1 provides a complete description of participants. Most (90.5%) held a bachelor’s degree or lower, whereas a small proportion (9.5%) held a master’s degree. The oldest age groups (≥ 50 years) and the youngest age group (≤ 29 years) were small in numbers. There were 128 (42%) females and 177 (58%) males, with a female-to-male ratio of 1:1.38. More than half had more than 10 years of experience (79.3%) and did not work in general practice (85.9%). A total of 176 (57.7%) participants worked in tertiary hospitals. A minority (n = 129, 42.3%) had no clinical teaching experience.

**Table 1:** Participant characteristics (n = 305)

Variable	Category	n	%
Age group (years)			
	-29	7	2.3
	30–39	180	59.0
	40–49	110	36.1
	50+	8	2.6
Sex			
	Male	177	58.0
	Female	128	42.0
Years of practice (years)			
	< 4	12	3.9
	5–9	48	15.7
	≥ 10	242	79.3
Education Level			
	No master’s degree	276	90.5
	Master’s degree	29	9.5
Workplace			
	Tertiary hospital	176	57.7
	Secondary hospital	129	42.3
Working in general practice			
	Yes	43	14.1
	No	262	85.9
Clinical teaching experience			
	Yes	176	57.7
	No	129	42.3

### Essential teaching knowledge

**Fig. 2:**
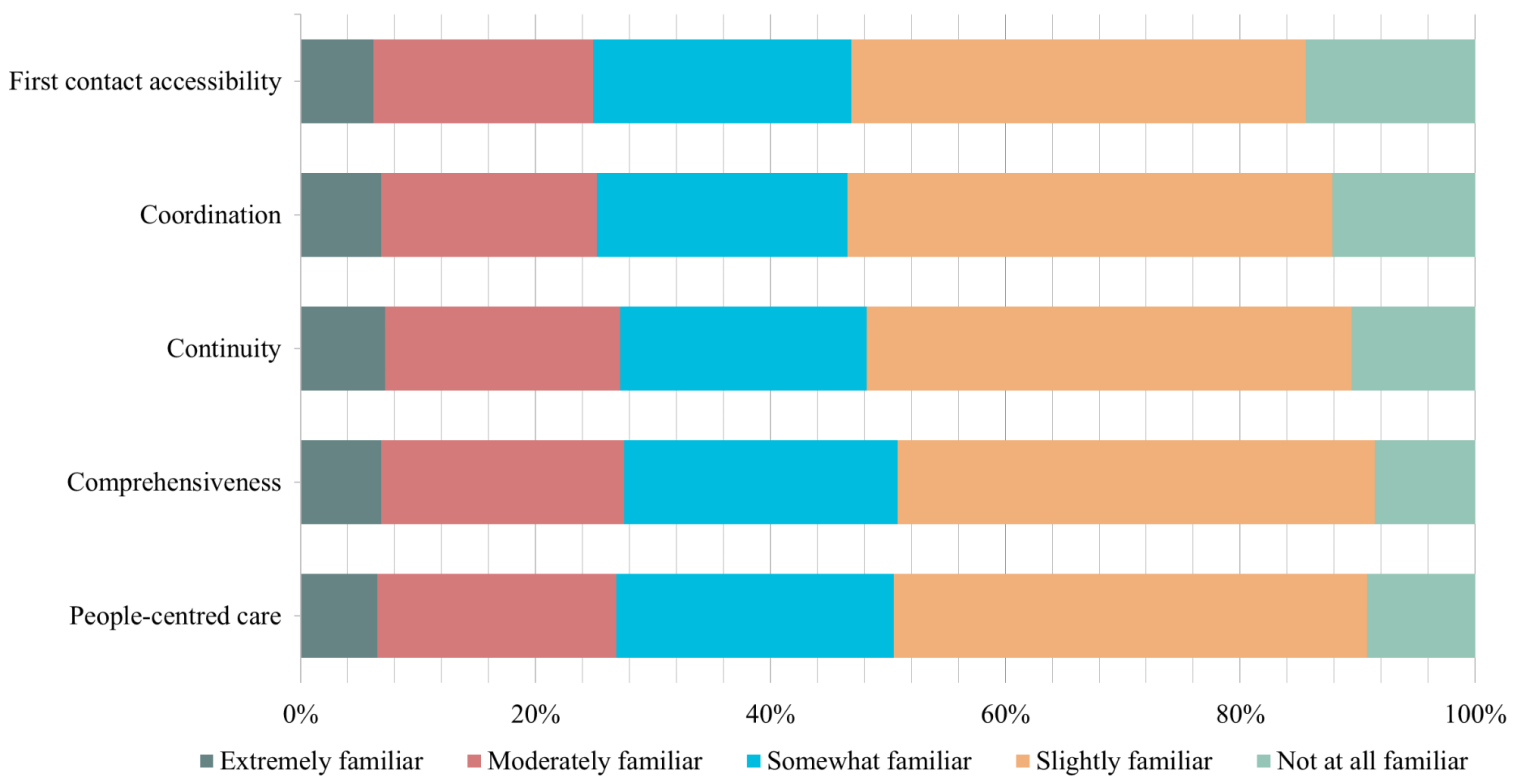
Familiarity levels in core functions of primary care skills

**Fig. 3:**
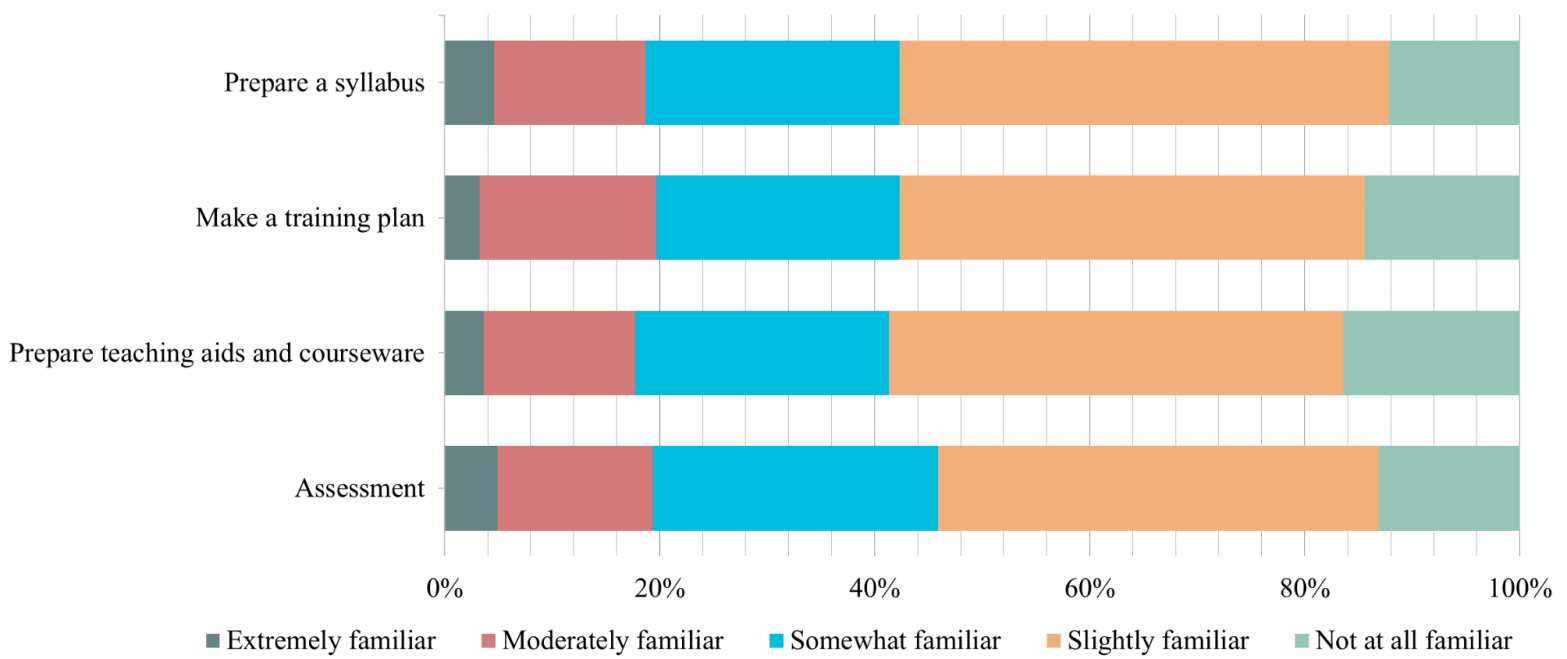
Familiarity levels in skills for preparation of lesson plan

**Fig. 4:**
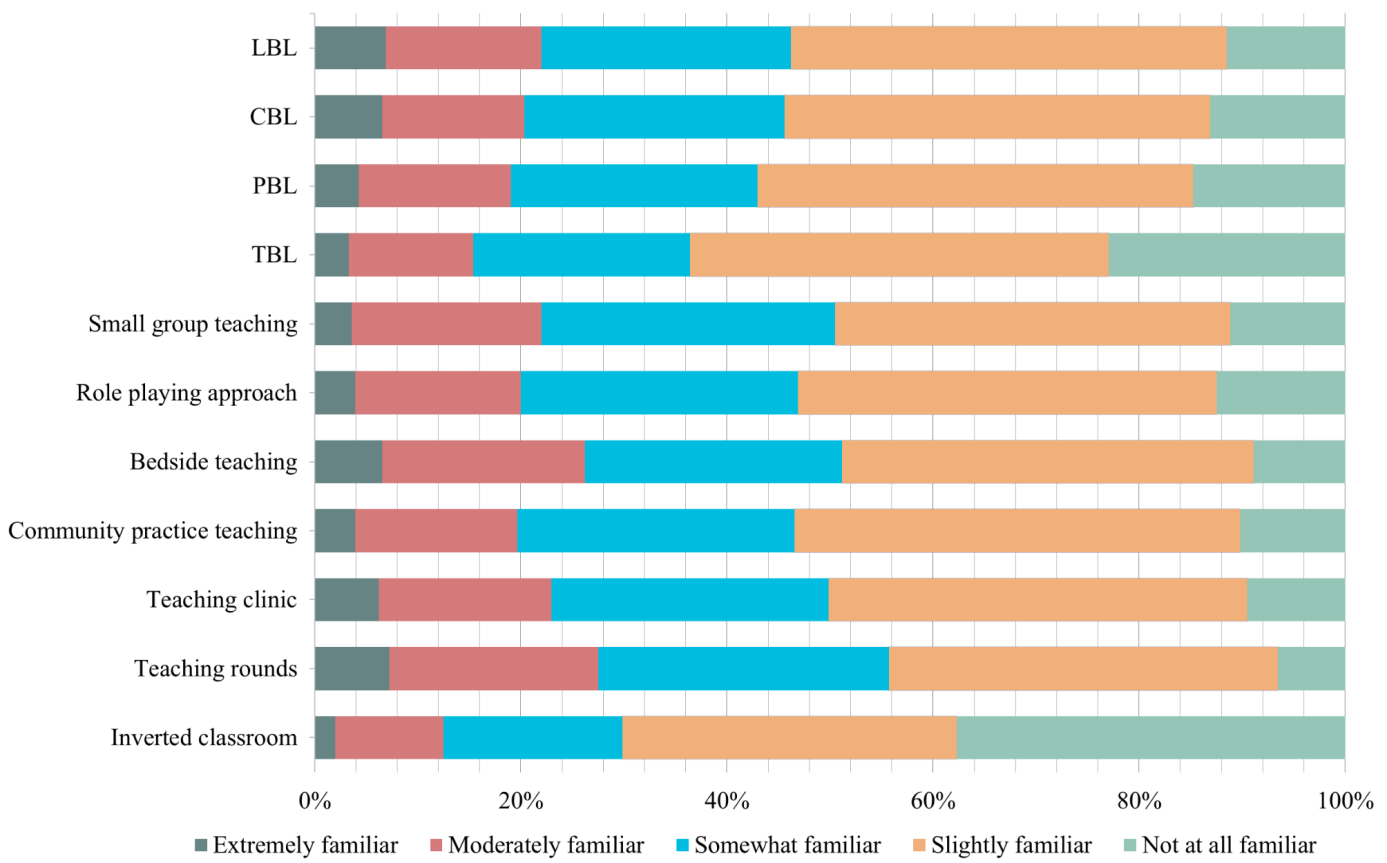
Familiarity levels in teaching method skills *Note*: CBL, case-based learning; PBL, problem-based learning; TBL, team-based learning; LBL, lecture-based teaching

The Cronbach’s alpha coefficients for the core functions of primary care, preparation for lesson plans, and teaching methods were 0.985, 0.967, and 0.972, respectively. In all skills, the proportion of respondents who selected “extremely familiar” was the smallest. Figures 2, 3 and 4 provided information on the relative familiarity with each item. Regarding the core functions of primary care, the largest number of respondents indicated slight familiarity for each item (Fig. 2). 18 (5.9%) reported being extremely familiar with the area. The skill with the largest number of respondents (14.4%) indicating “not at all familiar” was first contact accessibility, whereas only 6.2% indicated being extremely familiar with it. Among the four skills of preparation for the lesson plan, the highest proportion of respondents who rated “Prepare teaching aids and courseware” as “not at all familiar” was 16.4%, followed by “Make a training plan” (14.4%) and “Assessment” (13.1%). Only four respondents were familiar with all the four items (Fig. 3). Regarding teaching methods, the least five unfamiliarity skills were observed for teaching rounds at 6.6%, followed by bedside teaching at 8.9%, teaching clinics at 9.5%, community practice teaching at 10.2%, and small group teaching at 11.15%. Notably, the largest number of people (37.7%) who were not at all familiar with the inverted classrooms was reported, while only 2.0% (6) chose it as “extremely familiar” (refer to Fig. 4).

### Familiarity

Among the core functions of primary care, the three lowest rates of familiarity were reported for three essential skills: people-centred care (26.9%), coordination (25.2%), and first-contact accessibility (24.9%). Regarding lesson plan preparation, the least familiar skills were assessment (19.3%), syllabus preparation (18.7%), and preparation of teaching aids and courseware (17.7%). The most familiar teaching methods were teaching in clinics (23.0%), bedside teaching (26.2%), and teaching rounds (27.5%). No significant differences were observed in familiarity rates between tertiary and secondary hospitals (Table 2).

**Table 2:** Comparison of familiarity rates in tertiary and secondary hospitals

	All doctors, No. (%)	Doctors in tertiary hospitals, No. (%)	Doctors in secondary hospitals, No. (%)	χ^2^	*P*-value
Core functions of primary care					
People-centred care	82(26.9)	42(23.9)	40(31.0)	1.933	0.164
Comprehensiveness	84(27.5)	45(25.6)	39(30.2)	0.812	0.368
Continuity	83(27.2)	46(26.1)	37(28.7)	0.244	0.622
Coordination	77(25.2)	40(22.7)	37(28.7)	1.399	0.237
First contact accessibility	76(24.9)	40(22.7)	36(27.9)	1.067	0.302
Preparation for lesson plan					
Prepare a syllabus	57(18.7)	34(19.3)	23(17.8)	0.109	0.742
Make a training plan	60(19.7)	36(20.5)	24(18.6)	0.161	0.688
Prepare teaching aids and courseware	54(17.7)	33(18.8)	21(16.3)	0.312	0.576
Assessment	59(19.3)	32(18.2)	27(20.9)	0.360	0.548
Teaching method					
LBL	67(22.0)	37(21.0)	30(23.3)	0.217	0.642
CBL	62(20.3)	34(19.3)	28(21.7)	0.262	0.609
PBL	58(19.0)	31(17.6)	27(20.9)	0.532	0.466
TBL	47(15.4)	26(14.8)	21(16.3)	0.130	0.719
Small group teaching	67(22.0)	37(21.0)	30(23.3)	0.217	0.642
Role playing approach	61(20.0)	31(17.6)	30(23.3)	1.481	0.224
Bedside teaching	80(26.2)	48(27.3)	32(24.8)	0.234	0.629
Community practice teaching	60(19.7)	32(18.2)	28(21.7)	0.585	0.444
Teaching clinic	70(23.0)	38(21.6)	32(24.8)	0.435	0.509
Teaching rounds	84(27.5)	49(27.8)	35(27.1)	0.019	0.891
Inverted classroom	38(12.5)	19(10.8)	19(14.7)	1.056	0.304

*Note*: LBL, lecture-based teaching; CBL, case-based learning; PBL, problem-based learning; TBL, team-based learning

## Discussion

This study aimed to assess the baseline knowledge of GP transfer training trainers regarding their teaching skills in transfer training. Our results demonstrated that despite the majority of trainers having clinical teaching experience, they exhibited limited familiarity with the core functions of primary care, preparation for lesson plans, and teaching methods. This indicates significant gaps in their understanding of essential teaching skills and highlights the necessity for further training. These findings suggest that our study’s results are not unique to the context of China but rather reflect a broader necessity for training programmes for trainers worldwide.

One area in which our study identified significant knowledge gaps was the core function of primary care. Core functions are associated with better quality services, lower costs, less inequality in healthcare, and better population health [20]. This finding is particularly concerning, given the importance of these topics in primary care and their impact on patient outcomes. This may be caused by the absence of general practice-related courses offered during undergraduate education in China. Medical students may graduate without having learned the principles of general practice/family medicine or have not received relevant training after initiating clinical work. Research suggests that similar knowledge gaps regarding the core functions of primary care among trainers and specialists exist in other countries [21, 22]. Low awareness and lack of knowledge increase the barriers for trainers to integrate skills in daily practice [23, 24].

Our findings suggest that drawing up independent learning plans may be challenging for GPs trainers, particularly novices. Previous studies have highlighted the importance of effective lesson planning in training, including clearly defining learning objectives, selecting appropriate teaching materials, and providing feedback to peers and trainees [25–27]. Trainers may encounter difficulties generating planning decisions that align with the specific learning needs of their students [28]. The impact of short-term teaching programmes on GP trainers’ teaching ability of GPs trainers requires further study.

Knowledge gaps are pervasive across hospitals. Short courses should focus on easily mastered and readily preparable teaching methods, such as bedside teaching, teaching clinics, and teaching rounds, which have demonstrated effectiveness in medical education and are becoming increasingly standardised and homogenised in China [17, 29, 30]. Previous studies have shown that GPs prefer bedside teaching and intensive courses [15], which should be trained first because of the limited training time.

It is evident that the current GP transfer training in China falls short of adequately preparing trainers. One major issue is the lack of in-depth participation by general practice teaching teams in training programmes [14]. Although short-term training experience can reach a large number of trainers, it may lead to uneven faculty quality. Therefore, it is crucial to improve the training system and assessment standards for GPs trainers, explore effective training models, and enhance training quality. Policymakers must also consider whether these short continuing education programmes can improve trainers’ abilities [31].

The strengths of this study include the relatively large sample size and teaching skills assessment. To our knowledge, this study represents the first attempt to simultaneously evaluate the core functions of primary care, lesson plan preparation, and teaching methods among transfer training trainers in China. The present study has several limitations. The findings should be researched in studies with stronger methodological conceptions. First, self-assessed measures of confidence or competence may be weak surrogates for actual training needs and the self-report questionnaire may have limited objectivity. Future research should consider using objective indicators such as knowledge tests, objective structured clinical examinations (OSCE), and Direct Observation of Procedural Skills (DOPS). We are proceeding with our study plan. Second, the data are limited to Sichuan Province and therefore may not necessarily reflect practices in other regions. Similar training strategies have been implemented in other Chinese provinces. Third, the study only assessed baseline familiarity in three areas; the effectiveness of the training program was not evaluated. Further research should incorporate qualitative studies to explore trainers’ perspectives on the development and implementation of training programs. Information regarding whether participants were university faculty members was not collected, which was a limitation of this study and a necessity for future studies looking at teaching experience.

## Conclusions

In summary, our research revealed clear gaps in the core functions of primary care, preparation for lesson plans, and teaching methods among Chinese GP transfer training trainers. Addressing knowledge gaps is critical to ensure high-quality GP transfer training. Continuous training and motivation should be encouraged to enhance teaching competence further.

### Electronic supplementary material

Below is the link to the electronic supplementary material.

Supplementary Material 1

Supplementary Material 2

## Data Availability

All data and materials are available. The datasets generated for this study are available by contacting the corresponding author.

## Abbreviations

GP: General practitioner
LBL: Lecture-based teaching
CBL: Case-based learning
PBL: Problem-based learning
TBL: Team-based learning
