# Supplementary material for: Familiarity of teaching skills among general practitioners transfer training trainers in China: a cross-sectional survey
Source: BMC Med Educ. 2023 Dec 12;23:949. doi: 10.1186/s12909-023-04945-3 (PMC10717701; doi:10.1186/s12909-023-04945-3)
Supplement: Supplementary file 1 — Supplementary Material 1 [file 12909_2023_4945_MOESM1_ESM.docx]

**Overview of General Practitioners Transfer Training in China**

General practitioners (GPs) transfer training is a nationwide programme in China that aims to address the shortage of qualified GPs working in rural areas and community settings, improve the quality of primary care services, and reduce the burden on higher-level healthcare facilities [1, 2]. China has a substantial number of rural doctors, reaching 665,000 in 2022 [3], with the majority being non-GPs. Unfortunately, these doctors, although practicing in primary care, lack essential training in the principles of family medicine and general practice. This training targets clinical practitioners seeking to scope of practice transfer from other specialties to general practice. The target demographic includes two categories of clinical physicians. The first category consists of physicians are employed in primary healthcare institutions, such as rural doctors. The second category comprises specialist physicians working in hospitals. In both instances, these physicians have not undergone GP Transfer Training or participated in a standardized GP residency training program. Clinical rotation is an essential component of training and typically requires > 42 weeks [1]. It comprises general practice, internal medicine, and outpatient clinics for surgical, gynaecological, dermatological, and psychiatric specialties.

**References:**

1. National Health Commission of the People's Republic of China. The general practitioner transfer training syllabus (revised edition 2019). Available from: http://www.nhc.gov.cn/qjjys/s7945/201904/f0359ac60f714d5a82575a2f2155286a.shtml. (Accessed March 29,2022).

2. Lian S, Chen Q, Yao M, Chi C, Fetters MD. **Training Pathways to Working as a General Practitioner in China**. *Fam Med.* 2019; **51**(3):262-270. doi: 10.22454/FamMed.2019.329090.

3. National Health Commission of the People's Republic of China. Statistical Bulletin on the Development of Health and Health Care in China 2022. Available from: https://www.gov.cn/lianbo/bumen/202310/content_6908685.htm. (Accessed November 15, 2023).
