# Supplementary material for: Familiarity of teaching skills among general practitioners transfer training trainers in China: a cross-sectional survey
Source: BMC Med Educ. 2023 Dec 12;23:949. doi: 10.1186/s12909-023-04945-3 (PMC10717701; doi:10.1186/s12909-023-04945-3)
Supplement: Supplementary file 2 — Supplementary Material 2 [file 12909_2023_4945_MOESM2_ESM.docx]

**A TEACHING SKILLS QUESTIONNAIRE FOR GENERAL PRACTITIONERS TRANSFER TRAINING**

1. **Demographic Information**
2. Age group (years):
3. -29
4. 30-39
5. 40-49
6. 50+
7. Sex:
8. Male
9. Female
10. Year of practice (years):
11. <4
12. 5-9
13. ≥10
14. What is your education Level?
15. No master's degree
16. Master's degree
17. What type of Hospital do you work in?
18. Tertiary hospital
19. Secondary hospital
20. Do you work in general practice?
    1. Yes
    2. No
21. Do you have clinical teaching experience?
22. Yes
23. No
24. **Training contents**

In the following set of skills, we will inquire about level of familiarity in three essential teaching knowledge areas. On three Scale of 5-Eextremely familiar to 1-Not at all familiar, how do you assess skills in these areas?

**Part 1. Core functions of primary care**

| Skill | Not at all familiar | Slightly familiar | Somewhat familiar | Moderately familiar | Extremely familiar |
| --- | --- | --- | --- | --- | --- |
| People-centred care^a^ |  |  |  |  |  |
| Comprehensiveness^b^ |  |  |  |  |  |
| Continuity^c^ |  |  |  |  |  |
| Coordination^d^ |  |  |  |  |  |
| First contact accessibility^e^ |  |  |  |  |  |

*Note*: a: People-centred care ensures that people have the education and support needed to make decisions and participate in their own care. b: Comprehensiveness ensures that a diverse range of promotive, protective, preventive, curative, rehabilitative, and palliative services are provided. c: Continuity promotes the development of long-term personal relationships between a person and a health professional or a team of providers. d: Coordination organizes services and care across levels of the health system and over time. e: First contact accessibility creates a strategic entry point for and improves access to health services. *Reference*: World Health Organization. Primary care. Available from: https://www.who.int/teams/integrated-health-services/clinical-services-and-systems/primary-care.

**Part 2. Preparation for lesson plans**

| Skill | Not at all familiar | Slightly familiar | Somewhat familiar | Moderately familiar | Extremely familiar |
| --- | --- | --- | --- | --- | --- |
| Prepare a syllabus |  |  |  |  |  |
| Make a training plan |  |  |  |  |  |
| Prepare teaching aids and courseware |  |  |  |  |  |
| Assessment |  |  |  |  |  |

| **Part 3. Teaching method** |
| --- |

| Skill | Not at all familiar | Slightly familiar | Somewhat familiar | Moderately familiar | Extremely familiar |
| --- | --- | --- | --- | --- | --- |
| lecture-based teaching |  |  |  |  |  |
| case-based learning |  |  |  |  |  |
| problem-based learning |  |  |  |  |  |
| team-based learning |  |  |  |  |  |
| Small group teaching |  |  |  |  |  |
| Role playing approach |  |  |  |  |  |
| Bedside teaching |  |  |  |  |  |
| Community practice teaching |  |  |  |  |  |
| Teaching clinic |  |  |  |  |  |
| Teaching rounds |  |  |  |  |  |
| Inverted classroom |  |  |  |  |  |
